# The Dynamical Evolution Parameter in Manifestly Covariant Quantum Gravity Theory

**DOI:** 10.3390/e27060604

**Published:** 2025-06-05

**Authors:** Claudio Cremaschini

**Affiliations:** Research Centre for Theoretical Physics and Astrophysics, Institute of Physics, Silesian University in Opava, Bezručovo nám.13, CZ-74601 Opava, Czech Republic; claudiocremaschini@gmail.com

**Keywords:** covariant quantum gravity, Hamiltonian theory, evolution parameter, cosmological constant, 04.20.Cv, 04.20.Fy, 04.60.-m, 04.60.Ds, 11.10.Ef, 98.80.Qc

## Abstract

A remarkable feature of manifestly covariant quantum gravity theory (CQG-theory) is represented by its unconstrained Hamiltonian structure expressed in evolution form. This permits the identification of the corresponding dynamical evolution parameter advancing the quantum-wave equation for the 4−scalar quantum wave function defined on an appropriate Hilbert space. In the framework of CQG-theory, such a temporal parameter is represented by a 4−scalar proper time *s* identifying a canonical variable with conjugate quantum operator. The observable character of the evolution parameter is also established through its correspondence with the quantum representation of the cosmological constant originating from non-linear Bohm quantum–vacuum interaction, which is shown to admit an intrinsic functional dependence on *s*. These conclusions overcome the conceptual limitations about the so-called “problem of time” mentioned in alternative approaches to quantum gravity available in the literature. Hence, the outcome permits one to promote CQG theory as a viable mathematical setting for the establishment of a theory of quantum gravity consistent with the logical and physical principles of both general relativity and canonical quantum mechanics.

## 1. Introduction: The Problem of Time

A cornerstone of theoretical physics and field theory consists in the property that any Hamiltonian theory should admit a representation in evolution form, which will be achieved in terms of Hamilton equations expressed with respect to a suitable dynamical parameter. Based on the formalism developed in non-relativistic settings where the latter variable coincides with the absolute time, the natural identification in case of relativistic frameworks is usually in terms of the coordinate time *t* applying for appropriate sets of reference frames. Historically, this requisite was also implemented for classical general relativity (GR) itself and led to the establishment of the canonical Hamiltonian formulation of GR known as Arnowitt–Deser–Misner (ADM) formalism [1,2]. The correct identification of a physically meaningful evolution parameter in classical and quantum Hamiltonian theories of gravitational field and its physical interpretation represents challenging subjects of investigation in theoretical physics and cosmology, and formidable philosophical issues of debate demanding compelling answers [3,4,5,6]. This issue, together with the many attempts to find a solution that have been proposed over the years, provide the reference setting of the so-called “problem of time” of quantum gravity [7]. The target of this paper is to prove how a coherent solution to it can be achieved in the framework of manifestly covariant classical and quantum gravity theories (respectively denoted as CCG theory and CQG theory) [8,9]. Notice, however, that this paper is not intended as a review of the concept of time in quantum gravity. Extensive and exhaustive discussions on the issue can be found in relevant works in the literature, like in ref. [10]. For this reason, here, the focus is mainly on some relevant conceptual remarks that are specifically needed in support of the physical content of the research.

Let us start with a summary about the nature of the problem. The ADM approach is based on the mathematical formalism developed by Dirac to treat Hamiltonian field theory; in particular, the identification of canonical momenta in terms of coordinate-time partial derivative of metric tensor [11,12] and the Dirac theory of constrained dynamics [13]. Accordingly, the variational metric tensor $g_{\mu \nu }\left(r\right)$ is assumed to belong to a constrained functional setting and is represented in terms of the set of non-4−tensor ADM Lagrangian variables $\left(h_{ab}\left(r\right),N\left(r\right),N_{a}\left(r\right)\right)$, where $h_{ab}$ is the 3×3 variational matrix, while *N* and $N^{a}$ are, respectively, the “lapse” function defining the coordinate time and the “shift” 3−vector. Herein, the Latin indices a,b run from 1 to 3, while the Greek ones range, as usual, from 0 to 3. Hence, the use of ADM variables amounts to introducing a foliation of the 4−dimensional space-time into 3−space surface sections $\Sigma_{t}\equiv h_{ab}\left(r\right)$, each one of a constant coordinate–time *t*. Starting with the ADM Lagrangian density and upon introducing a Legendre transform in terms of momenta $p^{ab}$ conjugated to $h_{ab}$ yields a corresponding Hamilton variational principle for the set of ADM canonical variables $G\left(r\right)\equiv \left\{h_{ab},p^{ab},N,N_{a}\right\}$. The Euler–Lagrange equations that follow from it are represented by two Hamilton-like evolution equations for the conjugate variables $\left(h_{ab},p^{ab}\right)$, with the additional contribution of a 3−scalar and a 3−vector constraint equations. The latter ones are expressed as vanishing conditions of the form $H_{⊥}=0$ and $H^{a}=0$, where the “Hamiltonian” functions $H_{⊥}$ and $H^{a}$ are associated, respectively, with the lapse and shift functions *N* and $N_{a}$ [14].

The ADM Hamiltonian formulation has become a reference theory for the target of reaching a covariant quantization of GR and has represented a plausible candidate framework for the theoretical investigation and comprehension of quantum gravitational field. However, it was proved that the same ADM formalism exhibits critical mathematical and logical aspects of Hamiltonian field theory, also in connection with foundational principles of the covariant representation of GR. Additional limitations include the problems inherent to the same definition of Hamiltonian structure for ADM variables and the fact that ADM formalism belongs to a constrained field theory; see, for example, ref. [15]. Altogether, thus far, these features of the ADM setting have inhibited any definite progress or successful attempt toward the complete achievement of a corresponding canonical theory of quantum gravity. In this respect, a particularly crucial aspect of such an approach is the so-called “problem of time”, partly inherited from the classical ADM Hamiltonian formulation [10].

In fact, the problem arises from the validity of the Hamiltonian constraint $H_{⊥}=0$ in the passage to canonical quantization for the prescription of the quantum-gravity wave equation through the so-called Wheeler–deWitt (WdW) prescription [16]. In detail, this is achieved by promoting the Hamiltonian $H_{⊥}$ to be an operator, namely letting $H_{⊥}\to {\hat{H}}_{⊥}$, and then requiring ${\hat{H}}_{⊥}$ to act on the quantum wave function Ψ of the quantum gravitational field, yielding the WdW equation as

(1) $${\hat{H}}_{⊥}\Psi =0.$$

The last scalar equation, however, is not cast in 4−scalar form and therefore it does not represent an invariant relationship. Furthermore, it requires an appropriate regularization ordering scheme in order to handle the second-order functional derivatives appearing in the operator ${\hat{H}}_{⊥}$, whose prescription, however, is not unique within the theory. Overall, the form of the constraint WdW equation is purely stationary, without any time-dynamical content (with respect to the given time parameter) for the evolution of the wave function. In this sense, it is distinguished from a non-stationary Schroedinger-like wave equation in which the dynamical character of the quantum theory is made manifest through a clear identification of the evolution parameter.

On the contrary, in ADM formalism, the time coordinate is associated with a constraint equation yielding (1), so that at quantum level, the WdW constraint operator ${\hat{H}}_{⊥}$ becomes the generator of the dynamical evolution in the coordinate–time *t* of the 3+1 ADM foliation of space–time. Accordingly, in canonical quantum gravity, any operator representing a physical observable is required to commute with the same Hamiltonian operator ${\hat{H}}_{⊥}$, namely to be invariant under evolution with respect to the coordinate–time *t* [17]. This condition identifies the so-called Dirac observables and is at the root of the “problem of time”, so that any physical state corresponding to a Dirac observable is necessarily coordinate–time-independent in the same ADM reference frame. This apparently generates a purely stationary quantum state in which the usual notion of time evolution is trivial [18].

More generally, a deeper issue arises as to whether time itself might be considered an observable in canonical quantum gravity [18]. This would require the time to identify a canonical variable represented in terms of a self-adjoint operator having conjugate canonical momentum. However, from the conceptual foundations of quantum mechanics where there is no operator associated with time, an analogous conclusion can be inferred also for the coordinate-time of the constrained ADM quantum gravity [7]. In the past literature, different proposals were suggested with the aim of explaining the nature of the problem of time and opening new routes to try to find a solution to it. A review of the matter can be found in ref. [10]. Among them, the following two approaches are relevant for the connection with the present research:

(1) The first one is based on the idea of “relational evolution” [19,20], which applies in principle both at classical and quantum ADM frameworks and in the related representation referred to as loop quantum gravity. Accordingly, physical observables would not evolve dynamically with respect to a given temporal parameter, but rather the dynamics itself arises as an independent concept from their mutual relational evolution. A particular example of observables belonging to this group is provided by the “evolving constants of motion”, which include the quantum Dirac observables and which generate dynamics through relational evolution among tensor fields described by appropriate field equations [18].

(2) The second idea proposes to overcome the problem of time generated by the constraint WdW equation by constructing an unconstrained Hamiltonian [21] and implementing for classical and quantum gravity a formalism originally developed by Fock and Stueckelberg for relativistic particle dynamics. The goal of this method is to relax the Hamiltonian constraints and determine an unconstrained dynamical evolution through a method analogous to the time-dependent Schroedinger equation. The procedure generally generates a wider class of dynamical equations and solutions, requiring imposition of the constraints a posteriori. Attempts to implement this technique were proposed in scenarios of quantum cosmology for simplified models of the Universe. This is also exemplified by alternative formulations of GR known as “unimodular gravity” [21], in which the cosmological constant (CC) Λ is promoted to be a dynamical field subject to initial-value dynamics [22,23], in analogy to the non-constant relativistic particle rest mass in the Fock–Stueckelberg original theory. In such a setting, an effective cosmological time can be identified with the variable conjugate to the non-constant Λ [24].

Given these premises, it is possible to state that the problem of time can be effectively split into two main branches. The first one consists in the identification of the evolution parameter that must arise consistently from the canonical quantization of GR from its classical Hamiltonian structure. The second one deals with the possibility of assigning the same dynamical parameter a clear physical interpretation, e.g., possibly in terms of an observable (classical/quantum) field. However, in order to provide a novel point of view on the subject, a fundamental preliminary clarification must be obtained. This concerns the distinction between the concept of time as the non-invariant evolution parameter identified with the coordinate-time of a particular reference frame, and the dynamical parameter of temporal evolution to be possibly realized by an invariant field, therefore not coinciding with the coordinate-time.

The formal and conceptual limitations that arise from the literature concerning the problem of time rooted on the ADM formalism demand a change of strategy to gain some hopes of a solution, as progress in this direction remains inhibited in the ADM framework. In contrast, in the following, we proceed to prove that a possible consistent solution to the problem of time can be established in the frameworks of manifestly covariant CCG and CQG theories. The latter realize unconstrained Hamiltonian formulations of classical GR and its canonical quantum representation, consistent with the principle of manifest covariance of the deDonder–Weyl variational formalism of field theory [25,26,27,28,29,30,31,32,33,34]. A peculiar feature of CCG and CQG theories lies in their intrinsic evolution–form representations, which permit the identification of the temporal parameter and allow us to point out its physical properties, including its mathematical relationship with the cosmological constant. In turn, the theory also allows one to realize the concepts of unimodular gravity and relational evolution for CQG theory.

## 2. Classical Setting: CCG Theory

Let us start from the classical setting. The formalism of CCG-theory is characterized by the Lagrangian path parametrization of the Hamiltonian state based on the notion of Lagrangian path (LP) [35]. For this purpose, let us denote by ${\hat{g}}_{\mu \nu }$ the background metric tensor solution of the Einstein field equations, and by ${\hat{\nabla }}_{\alpha }$ the corresponding covariant derivative operator defined in terms of Christoffel symbols. Then, we introduce the real 4−tensor $t^{\gamma }\left(\hat{g}\left(r\right),r\right)$ which is tangent to an arbitrary geodetics at 4−position $r\equiv \left\{r^{\mu }\right\}$ of the space-time $\left(Q^{4},\hat{g}\left(r\right)\right)$ [36], namely

(2) $$\left\{\begin{array}{c} \;\;t^{\alpha }\left(\hat{g}\left(r\right),r\right){\hat{\nabla }}_{\alpha }t^{\gamma }\left(\hat{g}\left(r\right),r\right)=0,\\ \;\;{\hat{g}}_{\gamma \delta }\left(r\right)t^{\gamma }\left(\hat{g}\left(r\right),r\right)t^{\delta }\left(\hat{g}\left(r\right),r\right)=1. \end{array}\right.$$

The LP is identified with the geodetic curve

(3) $$\left\{r^{\mu }\left(s\right)\right\}\equiv \left\{\left.r^{\mu }\left(s\right)\right|\;\forall s\in R,\;r^{\mu }\left(s_{o}\right)=r_{o}^{\mu }\right\},$$

which is solution of the initial-value problem

(4) $$\left\{\begin{array}{c} \frac{dr^{\mu }\left(s\right)}{ds}=t^{\mu }\left(s\right),\\ r^{\mu }\left(s_{o}\right)=r_{o}^{\mu }. \end{array}\right.$$

Here, the notation is such that $t^{\mu }\left(s\right)\equiv t^{\mu }\left(\hat{g}\left(r\left(s\right)\right),r\left(s\right)\right)$, while *s* is the 4−scalar proper time defined along the same curve $\left\{r^{\mu }\left(s\right)\right\}$ by means of the differential identity $ds^{2}={\hat{g}}_{\mu \nu }\left(r\right)dr^{\mu }\left(s\right)dr^{\nu }\left(s\right)$, and $\frac{d}{ds}\equiv \frac{\partial }{\partial s}$ identifies the ordinary derivative with respect to *s*. The proper time parametrization in terms of *s* is then obtained, replacing in all the relevant tensor fields, $r\equiv \left\{r^{\mu }\right\}$ with $r\left(s\right)\equiv \left\{r^{\mu }\left(s\right)\right\}$.

In such a setting, the problem of obtaining a representation of the manifestly covariant Hamilton equations in evolution form is met in terms of a reduced-continuum Hamiltonian theory for GR. The latter is represented by the classical Hamiltonian system $\left\{x_{R},H_{R}\right\}$ formed by a 4−tensor canonical state $x_{R}$ and a 4−scalar Hamiltonian $H_{R}\left(x_{R},{\hat{x}}_{R}\left(r\right),r,s\right)$. The same Hamiltonian structure must generate continuum Hamilton equations to be cast in canonical evolution form, namely corresponding to the initial-value problem

(5) $$\left\{\begin{array}{c} \frac{Dg_{\mu \nu }\left(s\right)}{Ds}=\frac{\partial H_{R}\left(x_{R},{\hat{x}}_{R}\left(r\right),r,s\right)}{\partial \pi^{\mu \nu }\left(s\right)},\\ \frac{D\pi_{\mu \nu }\left(s\right)}{Ds}=-\frac{\partial H_{R}\left(x_{R},{\hat{x}}_{R}\left(r\right),r,s\right)}{\partial g^{\mu \nu }\left(s\right)}, \end{array}\right.$$

subject to the initial conditions of the type

(6) $$\left\{\begin{array}{c} g_{\mu \nu }\left(s_{o}\right)\equiv g_{\mu \nu }^{\left(o\right)}\left(s_{o}\right),\\ \pi_{\mu \nu }\left(s_{o}\right)\equiv \pi_{\mu \nu }^{\left(o\right)}\left(s_{o}\right). \end{array}\right.$$

The solution of the initial-value problem (5) and (6) associated with the Hamiltonian structure $\left\{x_{R},H_{R}\right\}$ generates the Hamiltonian flow

(7) $$x_{R}\left(s_{o}\right)\to x_{R}\left(s\right).$$

Here,

(8) $$x_{R}\left(s\right)\equiv \left\{g_{\mu \nu }\left(r\left(s\right)\right),\pi_{\mu \nu }\left(r\left(s\right)\right)\right\}$$

identifies the reduced-dimensional variational canonical state parametrized in terms of the proper time *s*, with $g_{\mu \nu }\left(r\right)$ and $\pi_{\mu \nu }\left(r\right)$ being the corresponding continuum Lagrangian coordinates and the conjugate momenta, ${\hat{x}}_{R}\left(s\right)\equiv \left\{{\hat{g}}_{\mu \nu }\left(r\left(s\right)\right),{\hat{\pi }}_{\mu \nu }\left(r\left(s\right)\right)\equiv 0\right\}$ being the corresponding prescribed state and $H_{R}\left(x_{R},{\hat{x}}_{R}\left(r\right),r,s\right)$ the variational 4−scalar Hamiltonian to be suitably determined. Finally, $\frac{D}{Ds}=\frac{\partial }{\partial s}+t^{\alpha }\left(s\right){\hat{\nabla }}_{\alpha }$ is the covariant s− derivative, while $t^{\alpha }\left(s\right)$ and ${\hat{\nabla }}_{\alpha }$ are, respectively, the tangent 4−vector to the geodesics $r\left(s\right)\equiv \left\{r^{\mu }\left(s\right)\right\}$ and the covariant derivative evaluated at the same position in terms of the background metric tensor ${\hat{g}}_{\mu \nu }\left(r\right)$.

In CCG theory, the reduced-dimensional Hamiltonian $H_{R}\left(x,\hat{x},s\right)\equiv H_{R}\left(x_{R},{\hat{x}}_{R}\left(r\right),r,s\right)$ is represented as

(9) $$H_{R}\left(x,\hat{x},s\right)\equiv \frac{1}{2}\frac{1}{\kappa }\pi_{\mu \nu }\pi^{\mu \nu }+\kappa h\left[g^{\mu \nu }{\hat{R}}_{\mu \nu }-2\Lambda \right],$$

where $\kappa =\frac{c^{3}}{16\pi G}$. In terms of the reduced Hamiltonian state, it is possible to develop a reduced Hamiltonian variational principle that yields Equation (5). To this end, the variational functional is identified with the real 4−scalar

(10) $$S_{R}\left(x,\hat{x}\right)\equiv \int d\hat{\Omega }L_{R}\left(x,\hat{x},r,s\right),$$

with $L_{R}\left(x,\hat{x},r,s\right)$ being the variational Lagrangian. This is related to the variational Hamiltonian $H_{R}\left(x,\hat{x},r,s\right)$ through the Legendre transform

(11) $$L_{R}\left(x,\hat{x},r,s\right)\equiv \pi_{\mu \nu }\frac{D}{Ds}g^{\mu \nu }-H_{R}\left(x,\hat{x},r,s\right).$$

Then, the variational principle that follows from the functional $S_{R}\left(x,\hat{x}\right)$ is prescribed by means of the synchronous unconstrained variational principle

(12) $$\delta S_{R}\left(x,\hat{x}\right)=0$$

obtained keeping constant both the state $\hat{x}$ and the 4−scalar volume element $d\hat{\Omega }$, where δ is the synchronous-variation operator expressed by the Frechet derivative according to ref. [8]. This yields the 4−tensor Euler–Lagrange equations that coincide with the Hamilton Equation (5) and that can be written in the equivalent Poisson-bracket representation as

(13) $$\frac{D}{Ds}x_{R}\left(s\right)={\left[x_{R},H_{R}\left(s\right)\right]}_{\left(x_{R}\right)},$$

with ${\left[,\right]}_{\left(x_{R}\right)}$ denoting the Poisson bracket evaluated with respect to the canonical variables $x_{R}$, namely

(14) $${\left[x_{R},H_{R}\left(s\right)\right]}_{\left(x_{R}\right)}=\frac{\partial x_{R}}{\partial g^{\mu \nu }}\frac{\partial H_{R}\left(s\right)}{\partial \pi_{\mu \nu }}-\frac{\partial x_{R}}{\partial \pi_{\mu \nu }}\frac{\partial H_{R}\left(s\right)}{\partial g^{\mu \nu }}.$$

In particular, invoking the explicit representation of $H_{R}\left(s\right)$ given by (9) gives

(15) $$\frac{\partial H_{R}\left(s\right)}{\partial g^{\mu \nu }\left(s\right)}=\kappa h\left(s\right){\hat{R}}_{\mu \nu }-\kappa g_{\mu \nu }\left(s\right)\frac{1}{2}\left(g^{\alpha \beta }\left(s\right){\hat{R}}_{\alpha \beta }\right),$$

where ${\hat{R}}_{\mu \nu }\equiv {\hat{R}}_{\mu \nu }\left(s\right)$ denotes the LP-parametrization of the Ricci tensor. The canonical Equation (5) then reduces to the single equivalent Lagrangian evolution equation for the variational field $g_{\mu \nu }\left(s\right)$:

(16) $$\frac{D}{Ds}\left[\frac{D}{Ds}g_{\mu \nu }\left(s\right)\right]+h\left(s\right){\hat{R}}_{\mu \nu }-g_{\mu \nu }\left(s\right)\frac{1}{2}\left[g^{\alpha \beta }\left(s\right){\hat{R}}_{\alpha \beta }-2\Lambda \right]=0.$$

The synchronous unconstrained Hamilton variational principle does not generate a single extremal field equation, but rather an entire class of solutions that vary depending on the set of initial values assigned to the canonical state. In this respect, the unconstrained synchronous variational approach might exhibit conceptual similarities with the relaxation of constraints approach and the unimodular gravity framework mentioned above, although it is established as an independent novel theory that keeps warranting the principle of manifest covariance. In particular, a fundamental requirement at this point of the derivation concerns the proof of the connection of the canonical Equation (5) with the Einstein theory of GR. This can be obtained under the assumption that the Hamiltonian does not depend explicitly on proper time *s*, i.e., it is actually of the form $H_{R}=H_{R}\left(x_{R},{\hat{x}}_{R}\left(r\right),r\right)$. Then, upon invoking the identities ${\hat{g}}_{\mu \nu }\left(s\right){\hat{g}}^{\mu \nu }\left(s\right)=\delta_{\mu }^{\mu }$ and $\frac{D}{Ds}{\hat{g}}_{\mu \nu }\left(s\right)\equiv 0$ holding for ${\hat{g}}_{\mu \nu }\left(s\right)$, it follows that ${\hat{\pi }}_{\mu \nu }\left(s\right)\equiv 0$, so that the canonical equation for ${\hat{\pi }}_{\mu \nu }\left(s\right)$ (or equivalently Equation (16)) finally yields for the background fields

(17) $${\hat{R}}_{\mu \nu }-{\hat{g}}_{\mu \nu }\left(s\right)\frac{1}{2}{\hat{g}}^{\alpha \beta }\left(s\right){\hat{R}}_{\alpha \beta }+\Lambda {\hat{g}}_{\mu \nu }\left(s\right)=0,$$

which coincides with the Einstein field equations. Therefore, in this framework, the latter arises as a stationary (with respect to proper time) solution to the GR-Hamilton Equation (5), i.e., imposing the initial conditions

(18) $$\left\{\begin{array}{c} g_{\mu \nu }\left(s_{o}\right)\equiv {\hat{g}}_{\mu \nu }\left(s_{o}\right),\\ \pi_{\mu \nu }\left(s_{o}\right)\equiv {\hat{\pi }}_{\mu \nu }\left(s_{o}\right)=0, \end{array}\right.$$

together with the requirement that for all s∈I

(19) $${\hat{\pi }}_{\mu \nu }\left(s\right)=0.$$

An interesting development of the Hamiltonian structure underlying CCG theory is provided by the possibility of also establishing the validity of Hamilton–Jacobi theory [8]. More precisely, the requirement is that the classical GR Hamilton Equation (13) is effectively equivalent to a single PDE of the type

(20) $$\frac{dS(g,\hat{g},r,s)}{ds}+H_{R}\left(g,\pi ,\hat{g},r,s\right)=0,$$

to be referred to as the GR–Hamilton–Jacobi equation. The latter holds for the 4−scalar Hamilton principal function $S(g,\hat{g},r,s)$ prescribed so that $\pi^{\mu \nu }=\frac{\partial S(g,\hat{g},r,s)}{\partial g_{\mu \nu }}$ identically. The mathematical proof of the equivalence between the GR-Hamilton equations and the GR–Hamilton–Jacobi equation, as well as the proof of consistency for the definition of the canonical momentum $\pi^{\mu \nu }$ in terms of $S(g,\hat{g},r,s)$ with the Hamiltonian structure of GR cast in evolution form, can be found in Theorem 1 of ref. [8]. This result represents a peculiar feature of the unconstrained manifestly covariant CCG functional setting with respect to constrained and non-manifestly covariant approach. Again, we notice that also in Equation (20) the dynamical parameter evolving the principal function $S(g,\hat{g},r,s)$ is the invariant proper time *s*, while both the 4−scalar functions $S(g,\hat{g},r,s)$ and $H_{R}\left(g,\pi ,\hat{g},r,s\right)$ can depend explicitly and implicitly on it, through the LP parametrization.

## 3. Quantum Setting: CQG Theory

Based on these results, CQG theory can then be obtained either by canonical quantization of the Hamiltonian structure or by adopting the quantization approach based on the equivalent Hamilton–Jacobi g−quantization starting from the classical Hamilton–Jacobi theory. Contrary to LQG/WdW theory based on ADM representation, the CQG state is identified with a single 4−scalar and complex function ψ(s) (CQG wave-function) of the form

(21) $$\psi \left(s\right)\equiv \psi (g,\hat{g}\left(r\right),r\left(s\right),s).$$

This corresponds to a spin-2 quantum particle with invariant rest mass $m_{o}>0$. In fact, according to the present approach, ψ(s) can be identified with the 4−scalar arising from the tensor product of the form $\psi \left(s\right)={\hat{g}}_{\mu \nu }\psi^{\mu \nu }$. Regarding the functional setting of ψ(s), it is assumed that it depends smoothly on the tensor field $g\equiv \left\{g_{\mu \nu }\right\}$ spanning the configurations space $U_{g}$, on the background field $\hat{g}\left(r\left(s\right)\right)\equiv \left\{{\hat{g}}_{\mu \nu }\left(r\left(s\right)\right)\right\}$, on the s−parametrized geodetics $r\left(s\right)\equiv \left\{r^{\mu }\left(s\right)\right\}$ and explicitly also on the proper time *s* associated with the same geodetics. The function ψ defined by Equation (21) spans a Hilbert space $\Gamma_{\psi },$ i.e., a finite-dimensional linear vector space endowed with the scalar product

(22) $$\langle \psi_{a}|\psi_{b}\rangle \equiv \int_{U_{g}}d\left(g\right)\psi_{a}^{*}\left(g,\hat{g}\left(r\right),r\left(s\right),s\right)\psi_{b}\left(g,\hat{g}\left(r\right),r\left(s\right),s\right),$$

with $d\left(g\right)\equiv \prod_{\mu ,\nu =1,4}$$dg_{\mu \nu }$ denoting the canonical measure on $U_{g}$ and $\psi_{a,b}\left(s\right)\equiv \psi_{a,b}(g,\hat{g}\left(r\right),$r(s),s) being arbitrary elements of the Hilbert space $\Gamma_{\psi }$, where as usual, $\psi_{a}^{*}$ denotes the complex conjugate of $\psi_{a}$. Then, the real function $\rho \left(s\right)\equiv \rho (g,\hat{g}\left(r\right),r\left(s\right),s)$ prescribed as

(23) $$\rho \left(s\right)\equiv {\left|\psi (s)\right|}^{2}$$

identifies on the configuration space $U_{g}$ the quantum probability density function (CQG-PDF) of $g\equiv \left\{g_{\mu \nu }\right\}$ in the volume element d(g) belonging to the configuration space $U_{g}$, namely associated with the CQG-state.

The quantum Hamiltonian operator is prescribed in terms of the classical Hamiltonian function of evolution representation $H_{R}$. In order to warrant that the canonical momenta have the dimension of an action, it is necessary to introduce a dimensional normalization of the Hamiltonian structure, letting

(24) $$\left\{x_{R},H_{R}\right\}\to \left\{{\bar{x}}_{R},{\bar{H}}_{R}\right\},$$

where the classical dimensionally normalized Hamiltonian structure $\left\{{\bar{x}}_{R},{\bar{H}}_{R}\right\}$ is defined in terms of the canonical state ${\bar{x}}_{R}\equiv \left\{{\bar{g}}_{\mu \nu },{\bar{\pi }}_{\mu \nu }\right\}$ and the Hamiltonian ${\bar{H}}_{R}$. More precisely, ${\bar{g}}_{\mu \nu }\equiv g_{\mu \nu }$ and ${\bar{\pi }}_{\mu \nu }=\frac{\alpha L}{k}\pi_{\mu \nu }$ is the normalized conjugate momentum, *L* is a 4−scalar scale length, and α is a suitable dimensional 4−scalar, both defined in ref. [9]. Instead, ${\bar{H}}_{R}$ is defined as the real 4−scalar field

(25) $${\bar{H}}_{R}\left({\bar{x}}_{R},\hat{g},r,s\right)={\bar{T}}_{R}\left(\bar{g},\hat{g},r,s\right)+\bar{V}\left(\bar{g},\hat{g},r,s\right),$$

with ${\bar{T}}_{R}\left(\bar{g},\hat{g},r,s\right)\equiv \frac{{\bar{\pi }}^{\mu \nu }{\bar{\pi }}_{\mu \nu }}{2\alpha L}$ and $\bar{V}\left(\bar{g},\hat{g},r,s\right)\equiv h\alpha L\left[g^{\mu \nu }{\hat{R}}_{\mu \nu }-2\Lambda \right]$ being the normalized effective kinetic and potential terms. Then, given the classical GR–Hamiltonian structure $\left\{{\bar{x}}_{R},{\bar{H}}_{R}\right\}$, the canonical quantization rules in the context of CQG theory are based on the following CQG correspondence principle realized by the map

(26) $$\begin{array}{ccc} & & \left.g_{\mu \nu }\to g_{\mu \nu }^{\left(q\right)}\equiv g_{\mu \nu },\right. \end{array}$$

(27) $$\begin{array}{ccc} & & \left.\pi_{\mu \nu }\equiv \frac{\partial S(g,\hat{g},r,s;P)}{\partial g^{\mu \nu }}\to \pi_{\mu \nu }^{\left(q\right)}\equiv -i\hbar \frac{\partial }{\partial g^{\mu \nu }},\right. \end{array}$$

(28) $$\begin{array}{ccc} & & \left.p\equiv -\frac{\partial S(g,\hat{g},r,s;P)}{\partial s}\to p^{\left(q\right)}\equiv -i\hbar \frac{\partial }{\partial s},\right. \end{array}$$

(29) $$\begin{array}{ccc} & & \left.H_{R}\left(g,\frac{\partial S(g,\hat{g},r,s;P)}{\partial g},\hat{g}\left(s\right),r,s\right)\to H_{R}^{\left(q\right)},\right. \end{array}$$

where $x^{\left(q\right)}\equiv \left\{g_{\mu \nu }^{\left(q\right)},\pi_{\mu \nu }^{\left(q\right)}\right\}$ is the quantum canonical state and $\pi_{\mu \nu }^{\left(q\right)}$ is the quantum momentum operator. An analogous map then applies for the dimensionally reduced functions ${\bar{\pi }}_{\mu \nu }$ and ${\bar{H}}_{R}^{\left(q\right)}$, while by construction ${\bar{g}}_{\mu \nu }\equiv g_{\mu \nu }$. In particular, one finds that

(30) $${\bar{H}}_{R}\to {\bar{H}}_{R}^{\left(q\right)}={\bar{T}}_{R}^{\left(q\right)}\left(\bar{\pi }\right)+\bar{V},$$

where ${\bar{H}}_{R}^{\left(q\right)}$ is the CQG–Hamiltonian Hermitian operator with ${\bar{T}}_{R}^{\left(q\right)}\left(\bar{\pi }\right)$ being the “kinetic density” quantum operator

(31) $${\bar{T}}_{R}^{\left(q\right)}\left(\bar{\pi }\right)=\frac{\pi^{(q)\mu \nu }\pi_{\mu \nu }^{\left(q\right)}}{2\alpha L}.$$

The mapping realized by Equations (26)–(29) implies the simultaneous validity of the two fundamental commutator relations

(32) $$\left[g_{\mu \nu }^{\left(q\right)},\pi^{(q)\alpha \beta }\right]=i\hbar \delta_{\mu }^{\alpha }\delta_{\nu }^{\beta },$$

(33) $$\left[p^{\left(q\right)},s\right]=-i\hbar ,$$

together with

(34) $$\left[g^{\alpha \beta },g_{\mu \nu }\right]=\left[\pi^{(q)\alpha \beta },\pi_{\mu \nu }^{\left(q\right)}\right]=0.$$

The quantization rules (26)–(29) follow as a straightforward prescription within the canonical setting of deDonder–Weyl formalism of CQG theory, representing the tensorial generalization of the standard quantization method developed in non-relativistic quantum mechanics.

We can now proceed with prescribing the quantum-gravity wave equation that advances, in proper time, the quantum state ψ of CQG theory. We promote the Poisson brackets to quantum commutator; this is provided by the CQG-quantum wave equation

(35) $$i\hbar \frac{\partial }{\partial s}\psi \left(s\right)+\left[\psi \left(s\right),{\bar{H}}_{R}^{\left(q\right)}\right]=0,$$

where $\left[A,B\right]\equiv AB-BA$ denotes the quantum commutator, i.e.,

(36) $$\left[\psi \left(s\right),{\bar{H}}_{R}^{\left(q\right)}\right]\equiv -{\bar{H}}_{R}^{\left(q\right)}\psi \left(s\right).$$

The CQG wave Equation (35) prescribes the evolution of the quantum state ψ(s) along the geodetics of the background metric tensor ${\hat{g}}_{\mu \nu }\left(r\right)$. Equation (35) is a first-order partial differential equation that must be supplemented by prescribing for all $r\left(s_{o}\right)=r_{o}\in \left(Q^{4},\hat{g}\left(r\right)\right)$ the initial condition $\psi \left(s_{o}\right)=\psi_{o}\left(g,\hat{g}\left(r_{o}\right),r_{o}\right)$, as well as boundary conditions at infinity on the improper boundary of configuration space $U_{g}$, namely $\lim_{g\to \infty }\psi \left(g,\hat{g}\left(r\right),r\left(s\right),s\right)=0$.

The CQG-wave Equation (35) can be represented in terms of an equivalent set of quantum hydrodynamic equations [9]. This requires the adoption of the Madelung representation

(37) $$\psi \left(g,\hat{g},r,s\right)=\sqrt{\rho (g,\hat{g},r,s)}\exp\left\{\frac{i}{\hbar }S^{\left(q\right)}\left(g,\hat{g},r,s\right)\right\},$$

where the quantum fluid fields $\left\{\rho ,S^{\left(q\right)}\right\}\equiv \left\{\rho \left(g,\hat{g},r,s\right),S^{\left(q\right)}\left(g,\hat{g},r,s\right)\right\}$ identify, respectively, the 4−scalar quantum PDF and quantum phase function. Elementary algebra then shows that based on Equation (35) the same quantum fluid fields must satisfy the set of GR–quantum hydrodynamic equations (CQG-QHE) realized, respectively, by continuity and quantum Hamilton-Jacobi equations given by

(38) $$\begin{array}{ccc} \frac{d\rho }{ds}+\frac{\partial }{\partial g_{\mu \nu }}\left(\rho V_{\mu \nu }\right) & = & 0, \end{array}$$

(39) $$\begin{array}{ccc} \frac{dS^{\left(q\right)}}{ds}+H^{\left(q\right)} & = & 0, \end{array}$$

where in both equations $\frac{d}{ds}\equiv D_{s}$ [9]. Furthermore,$V_{\mu \nu }\equiv V_{\mu \nu }\left(g,s\right)$ and $H^{\left(q\right)}\equiv H^{\left(q\right)}\left(g,s\right)$ denote, respectively, the quantum 4−tensor velocity field identified with

(40) $$V_{\mu \nu }=\frac{1}{\alpha L}\frac{\partial S^{\left(q\right)}}{\partial g^{\mu \nu }},$$

and the effective quantum Hamiltonian

(41) $$H^{\left(q\right)}=\frac{1}{2\alpha L}\frac{\partial S^{\left(q\right)}}{\partial g^{\mu \nu }}\frac{\partial S^{\left(q\right)}}{\partial g_{\mu \nu }}+V_{QM}+\bar{V},$$

with $\bar{V}\equiv \bar{V}\left(g,s\right)$ being the effective potential defined above and $V_{QM}\equiv V_{QM}\left(g,s\right)$ being the Bohm effective quantum potential [37,38,39] given by

(42) $$V_{QM}\equiv \frac{\hbar^{2}}{8\alpha L}\frac{\partial \ln\rho }{\partial g^{\mu \nu }}\frac{\partial \ln\rho }{\partial g_{\mu \nu }}-\frac{\hbar^{2}}{4\alpha L}\frac{\partial^{2}\rho }{\rho \partial g_{\mu \nu }\partial g^{\mu \nu }}.$$

For completeness, it is worth noting that a precise relationship between the quantum and classical Hamilton–Jacobi Equations (39) and (20) as well as the one existing between quantum and classical tensor fields $V_{\mu \nu }$ and $\pi_{\mu \nu }$ can be established in terms of so-called semi-classical limit, whereby the Bohm quantum potential vanishes (see proof in ref. [9]).

We notice that the quantum gravity theory obtained here preserves the character of *s* to be the dynamical temporal parameter in CQG-theory. In addition, a remarkable feature is that *s* has the conjugate quantum operator $p^{\left(q\right)}\equiv -i\hbar \frac{\partial }{\partial s}$, a feature that is missing in the ADM formulation of the problem. This conclusion suggests that in such a framework, *s* must correspond to a physical observable, as will be proved below.

## 4. The Dynamical Evolution Parameter

Let us comment in more detail some remarks on the meaning of proper time in CQG theory. As shown above, the proper time *s* is associated with non-null subluminal geodetic curves of massive gravitons predicted by CQG theory on the space–time $\left\{Q^{4},\hat{g}\right\}$. The adoption of the proper time parametrization of classical and quantum Hamiltonian states permits us to recover the standard formalism of quantum mechanics for CQG-theory, which is related to the Hamiltonian structure of the theory and the physical interpretation of the quantum wave function. We notice that the choice of the 4−scalar proper time, contrary to the coordinate time, is consistent with the manifest covariance principle, while it preserves the role of temporal dynamical variable in terms of which dynamical evolution of quantum systems is parametrized. It follows that the notion of proper time becomes necessary for the representation of the fundamental equations of CQG theory and its physical interpretation. The invariant proper time is the dynamical parameter of CQG theory, to be distinguished from the coordinate time adopted, for example, in loop quantum gravity (for a discussion of this issue, see refs. [40,41]).

In fact, in the framework of background CQG theory, the metric tensor ${\hat{g}}_{\mu \nu }$ determines the differential Riemann distance ds on the space–time $\left\{Q^{4},\hat{g}\right\}$ and, consequently, also the line element (arc length) of proper time *s* by means of the 4−scalar equation

(43) $$ds^{2}={\hat{g}}_{\mu \nu }dr^{\mu }dr^{\nu },$$

where the 4−tensor displacement $dr^{\mu }$ around a 4−position $r\equiv \left\{r^{\mu }\right\}$ belongs to the subset of $\left\{Q^{4},\hat{g}\right\}$ where ${\hat{g}}_{\mu \nu }dr^{\mu }dr^{\nu }\geq 0$. By integration, it follows that

(44) $$s-s_{1}=\int_{r_{1}}^{r}\sqrt{{\hat{g}}_{\mu \nu }dr^{\mu }dr^{\nu }},$$

where, here, r≡r(s) and $r_{1}\equiv r\left(s_{1}\right)$ denote two 4−positions of the observer (i.e., the massive graviton) along an arbitrary curve r(s) joining them, while *s* and $s_{1}$ are the corresponding proper times. Such a worldline can be one of the (infinite possible) curves that cross the same 4−position $r^{\mu }$, i.e., an arbitrary observer’s geodetics $r\left(s\right)\equiv \left\{r^{\mu }\left(s\right)\right\}$ prescribed in such a way that at proper time s>0 it coincides with the observer’s position according to the initial (crossing) condition

(45) $$r^{\mu }=r^{\mu }\left(s\right).$$

However, after imposing (45), the precise value of *s* still depends both on the choice of the space–time curve on which it is measured and that of the reference 4−position $r_{1}=r\left(s_{1}\right)$ on the same curve. In fact, these geodetics are intrinsically non-unique, since for an arbitrary observer with 4−position $r\equiv \left\{r^{\mu }\right\},$ there are infinite geodesic curves $r\left(s\right)\equiv \left\{r^{\mu }\left(s\right)\right\}$ fulfilling the crossing condition (45). However, the notion of (observer) proper time (*s*) makes sense only if *s* is an observable, which demands a suitable way to prescribe it. In this respect, two choices are possible. In the first case, proper time is an observer proper time, i.e., a local observable which may nevertheless have different realizations for each observer (i.e., GR frames which are mutually connected via the local-point transformation group). Accordingly, the proper time *s* is, by construction, the same one for all geodesic trajectories which simultaneously cross the observer 4−position (see Equation (45)). The second possible realization is provided instead by the notion of global proper time, i.e., a global observable which is the same one for a family of observers which are properly “synchronized” with each other in such a way that the observer proper time *s* indeed coincides for all of them.

Let us analyze the conceptual basis for the prescriptions of the functional settings of the two choices separately. In the first case, a non-trivial definition of the observer proper time consistent with its identification with a graviton’s virtual worldline requires that

(1) For each observer, the corresponding geodesic curves (observer geodetics) are non-vanishing and have proper orientation.

(2) Since classical geodetics of particles with finite mass cannot cross event horizons, curves originating near them must have an origin point $r\left(s_{o}\right)=r_{o}$. This includes, in particular, the cosmological event horizon originated from the Big Bang event. It is understood that the indicated origin point $r\left(s_{o}\right)=r_{o}$ corresponds to a creation point of a graviton’s virtual worldline, which can lead to the assumption that all the observer’s geodesic curves have proper origin points and hence are semi-infinite. In addition, the origin points of all observer geodesic curves cannot belong to event horizons but can only be arbitrarily close to them, in the sense of mathematical limit.

(3) For all semi-infinite geodesic curves, it makes sense to require that the initial proper time $s_{o}$ is positive or null. For the uniqueness of *s* for a given observer, there must exist maximal geodetics, namely a geodesic curve with origin point $r^{\mu }\left(s_{o}\right)$ coinciding (or being suitably close) to the Big Bang event, having the maximal arc length $s-s_{o}$ and subject to the condition

(46) $$s_{o}=0,$$

with $s_{o}=0$ to be referred to as Big Bang proper time. A cosmological interpretation arises. Accordingly, such curves should have originated suitably near the universe horizon created during Big Bang, which is characterized by the lowest initial proper time. Thus, the root (46) identifies the proper-time of a (possibly virtual) graviton generated after the Big Bang event. The remaining trajectories which are associated with a given observer identify instead (again possibly virtual) massive gravitons which are generated at later proper times.

Instead, for the identification of the proper time *s* also as a global observable it is necessary to require, in addition, that

(4) For all observers which can be mutually connected by null geodetics (i.e., necessarily belong to the same light cone) and for all semi-infinite geodesic curves which are associated with them, the corresponding initial proper-times $s_{o}$ are all positive or null.

(5) Among them, for all observers, there is again for each one a “maximal length” possibly non-unique geodetic with the origin point $r^{\mu }\left(s_{o}\right)$, such that the condition (46) holds.

Two interpretations of proper time can be proposed at this point. The first one concerns the customary interpretation occurring in the context of general relativity [40,42], i.e., in terms of the Riemann distance on the space–time. This yields the so-called geometric interpretation, which is based on Equations (43) and (44). However, this does not provide, by itself, a unique prescription for *s*.In fact, once the reference 4−position r=r(s) (see Equation (45)) is prescribed, the precise value of *s* depends both on the choice of the space–time curve on which it is measured and that of the reference 4−position $r_{1}=r\left(s_{1}\right)$ on the same curve. As shown above, these indeterminacies can be resolved if, for all observers belonging to the same light cone, proper time is the arc length measured along arbitrary observer geodetics with origin point $r(s_{o})$ and in particular along an observer’s maximal geodetics with the origin point $r(s_{o}=0)$. The second possibility yields instead a dynamic interpretation. Thus, under the assumption of existence of massive gravitons, proper time can also acquire the further interpretation according to which, for all observers belonging to the same light-cone, it is the arc length of the worldline of a graviton measured between its origin point $r(s_{o}=0)$ and the observer position r(s)=r.

It is interesting to comment at this point about the crucial aspect of both CCG and CQG theories concerning the treatment of gravitons, i.e., the quanta of the gravitational field and in particular the related prescription of the notion of proper time (*s*). In fact, it must be stressed that both in CCG and CQG theories the background space–time ${\hat{g}}_{\mu \nu }$ prescribing the coordinate and the geometric properties of the reference system is not directly quantized as a single field. The quantization pertains the fluctuations with respect to ${\hat{g}}_{\mu \nu }$ of the quantum gravitational field described by $g_{\mu \nu }$, from which ${\hat{g}}_{\mu \nu }$ emerges as ensemble statistical average. The meaning of CQG theory is that of providing a spectrum of quantum $g_{\mu \nu }$ which altogether contribute to the emergence of the background tensor ${\hat{g}}_{\mu \nu }$. The quantum dynamics of $g_{\mu \nu }$ determines quantum effects to the solution of ${\hat{g}}_{\mu \nu }$ through quantum-modified Einstein field equations [9]. Accordingly, classical space–time solutions can inherit in this way the underlying quantum-gravity effects. Their inclusion in the Einstein equations however does not affect the basic conceptual structure of GR and the geometric interpretation of GR metric tensor. The implication is that, the dynamics of each quantum field $g_{\mu \nu }$ is governed by quantum-gravity wave equation when referred to the g− configuration space associated with the Hamiltonian structure $\left\{{\bar{x}}_{R},{\bar{H}}_{R}\right\}$. However, when referred to the physical background space–time associated with ${\hat{g}}_{\mu \nu }$ which defines the coordinate system, namely the generally non-inertial reference frame, gravitons still need to be treated as classical particles, i.e., as point-like neutral, spin-2 collisionless particles following classical geodesic curves on ${\hat{g}}_{\mu \nu }$. In fact, in order to quantize them, one should actually perform a full quantization of the metric tensor defining the space–time, and therefore the physical coordinates identified with position and velocity; however, this is not the target of CQG-theory. In addition, in view of the invariant discrete energy spectrum discovered in ref. [9] gravitons must carry a non-vanishing mass. Therefore, in the background metric tensor $\hat{g}$ they are endowed with a geodesic motion and their admissible worldlines must be identified with (deterministic) non-null subluminal geodetics. This is the reason why the same proper time evolution parameter *s* appears in both classical and quantum Hamiltonian theories of GR in such a framework.

The same kind of reasoning then applies to the concept of horizons invoked above to provide an interpretation of proper time. In fact, event horizons must be treated as classical surfaces when referred to the physical configuration space described by the background metric tensor ${\hat{g}}_{\mu \nu }$. This means that the same horizon surfaces arise from the solutions of the Einstein field equations. However, this approach does not exclude the possibility of having event horizons whose solution contains quantum-gravity effects. This can be reached by first determining quantum-gravity corrections or source terms to the classical Einstein field equations, yielding quantum-modified Einstein field equations, and then by solving them for the metric tensor. A solution method of this type has been carried out elsewhere in the framework of CQG theory for the treatment of both cosmological deSitter and black-hole event horizons [9].

## 5. Dynamical Parameter and Quantum Cosmological Constant

In terms of the previous results, the mathematical connection between the evolution parameter *s* and observable fields of classical and/or quantum gravity can be established. More precisely, the last part of the present research is devoted to pointing out the relationship that exists between the proper-time *s* and the cosmological constant Λ. This is achieved in the framework of CQG theory, whereby the quantum-generated cosmological constant is a 4−scalar that is found to vary on proper time *s* and to arise from the non-linear vacuum Bohm interaction among massive gravitons. This target permits to reach a number of notable results, namely:

(1) To elucidate the physical interpretation of the evolution proper time in terms of the observable cosmological constant.

(2) To assign the same parameter *s* the connotation of a cosmological proper time, namely a cosmological evolution parameter of the Hamiltonian theory.

(3) To link the quantum origin of the cosmological constant to the evolution parameter *s* introduced in the classical theory.

(4) To prove that from the physical point of view, the dynamical evolution of classical and quantum Hamiltonian systems is rooted on the quantum-gravity theory which predicts a proper time-varying cosmological constant. On the other hand, the same 4−scalar parameter also finds a mathematical definition in the setting of CCG-theory, proving its transversal role for both classical and quantum settings.

(5) To establish conceptual connections between fundamentals of CQG theory and other settings found in the literature, in particular concerning the so-called unimodular gravity theories and the relational concept underlying the theory of evolving constants of motion.

To introduce this discussion, we preliminarily recall the result achieved in ref. [43] concerning the classical variational theory of the cosmological constant and its consistency with quantum prescription. In the same work, in fact, it was proved that the manifestly covariant Hamiltonian structure of classical general relativity can be shown to be associated with a path-integral (rather than a configuration-space integral) synchronous Hamilton variational principle yielding the Einstein field equations. A realization of the same variational principle in both unconstrained and constrained forms can be established. The first case is based on the introduction of the Hamilton functional

(47) $$J\left(x_{R}\right)=\int_{s_{o}}^{s_{1}}ds\left(\pi^{\mu \nu }\left(s\right)\frac{dg_{\mu \nu }\left(s\right)}{ds}-H_{R}\left(x_{R}\left(s\right),s\right)\right),$$

and the synchronous variational principle

(48) $$\delta J(x_{R})=0,$$

with

(49) $$L_{R}\left(g\left(s\right),\frac{dg\left(s\right)}{ds},s\right)=\pi^{\mu \nu }\left(s\right)\frac{dg_{\mu \nu }\left(s\right)}{ds}-H_{R}\left(x_{R}\left(s\right),s\right)$$

denoting the Legendre-conjugate Lagrangian. The same variational principle delivers Euler–Lagrange equations in Hamiltonian form which coincide with the canonical Equation (5). In fact, the functional derivatives of $J(x_{R})$ yield explicitly

(50) $$\left\{\begin{array}{c} \frac{\delta J(x_{R})}{\delta \pi^{\mu \nu }}=\frac{dg_{\mu \nu }}{ds}-\frac{\partial H_{R}}{\partial \pi^{\mu \nu }}=0,\\ \frac{\delta J(x_{R})}{\delta g_{\mu \nu }}=-\frac{d\pi^{\mu \nu }}{ds}-\frac{\partial H_{R}}{\partial g_{\mu \nu }}=0, \end{array}\right.$$

with the solutions being subject to the boundary conditions

(51) $$x_{R}\left(s_{i}\right)=\left(g_{\mu \nu }\left(s_{i}\right),\pi^{\mu \nu }\left(s_{i}\right)\right),$$

in which, for i=0,1, the boundary tensor fields $g_{\mu \nu }\left(s_{o}\right)$ and $\pi^{\mu \nu }\left(s_{o}\right)$ remain, in principle, arbitrary. As in classical analytical mechanics, this shows that Hamilton equations for the SF-GR can be equivalently determined in terms of a Hamilton variational principle in which the variational functional is a path-integral of the form (47). This identifies a unique feature of the manifestly covariant synchronous approach, which allows to cast the variational functional as a line integral.

Nevertheless, a second equivalent possibility exists, in terms of a constrained Hamilton variational principle, to be realized by a path-integral functional performed again along a generic finite-length field geodetic $r\left(s\right)$. We adopt, for this purpose, the standard method of Lagrange multipliers, thus introducing the functional

(52) $$J_{L}\left(x_{R}\right)=J\left(x_{R}\right)+J_{1}\left(x_{R}\right)+J_{2}\left(x_{R}\right),$$

where, respectively, $J(x_{R})$ is defined by Equation (47), while

(53) $$J_{1}\left(x_{R}\right)=-\frac{1}{2}\int_{s_{o}}^{s_{1}}ds\lambda_{1}\left(g^{\mu \nu }g_{\mu \nu }-4\right),$$

(54) $$J_{2}\left(x_{R}\right)=-\frac{1}{2}\int_{s_{o}}^{s_{1}}ds\lambda_{2}\pi^{\mu \nu }\pi_{\mu \nu }.$$

Then, let us consider the constrained synchronous variational principle $\delta J_{L}\left(x_{R}\right)=0$ performed in terms of independent variations of the Lagrangian coordinates, the conjugate momenta and of the two Lagrange multipliers $\lambda_{1}$ and $\lambda_{2}$, while letting again δds=0 and $\delta \hat{g}=0$. The corresponding Euler–Lagrange equations are given, respectively, by the equations

(55) $$\left\{\begin{array}{c} \frac{\delta J_{L}\left(x_{R}\right)}{\delta \pi^{\mu \nu }}=\frac{dg_{\mu \nu }}{ds}-\frac{\partial H_{R}}{\partial \pi^{\mu \nu }}-\lambda_{2}\pi_{\mu \nu }=0,\\ \frac{\delta J_{L}\left(x_{R}\right)}{\delta g_{\mu \nu }}=-\frac{d\pi^{\mu \nu }}{ds}-\frac{\partial H_{R}}{\partial g_{\mu \nu }}-\lambda_{1}g^{\mu \nu }=0,\\ \frac{\delta J_{L}\left(x_{R}\right)}{\delta \lambda_{1}}=g^{\mu \nu }g_{\mu \nu }-4=0,\\ \frac{\delta J_{L}\left(x_{R}\right)}{\delta \lambda_{2}}=\pi^{\mu \nu }\pi_{\mu \nu }=0, \end{array}\right.$$

where the Lagrange multiplier $\lambda_{1}$ remains arbitrary.

Then, let us impose the identity $g_{\mu \nu }\equiv {\hat{g}}_{\mu \nu }$ holding for extremal curves. Thanks to the constraint equation associated with the undetermined Lagrange multiplier $\lambda_{2}$ (i.e., the fourth equation in the previous system), the condition of vanishing derivative $\frac{d{\hat{g}}_{\mu \nu }}{ds}=0$ provided by the first equation remains identically satisfied for all *s*. Furthermore, the second equation in particular delivers that a modified Einstein field equation actually holds, in which the contribution of the CC Λ is associated with the multiplier $\lambda_{1}$, namely upon letting

(56) $$\Lambda \equiv \lambda_{1}.$$

We notice that in the present context, $\lambda_{1}$ can be regarded as a classical parameter, which in general can be considered an arbitrary function of the form $\lambda_{1}=\lambda_{1}\left(\hat{g},r\left(s\right),s\right)$, consistent with the synchronous principle. Notice, however, that possible dependences of CC on $r\left(s\right)$ can be ruled out based on the symmetry property of the Einstein field equations. Similarly, possible dependences on $\hat{g}$ occurring through 4−scalar saturations of the Ricci or Riemann tensors remain excluded as they are not part of the standard Einstein field equations. The same Lagrange multiplier therefore must identify a 4−scalar gauge function generally dependent on the invariant proper time parameter *s* only, i.e., $\lambda_{1}=\lambda_{1}\left(s\right)$ ultimately.

The conclusion is that within the same context, at most, the CC is of the form

(57) $$\Lambda \equiv \Lambda \left(s\right).$$

As a consequence, the simultaneous validity of both unconstrained and constrained path-integral principles supports the interpretation of the cosmological constant in terms of a Lagrange multiplier associated with the normalization constraint for the extremal metric tensor $\hat{g}\equiv \left\{{\hat{g}}_{\mu \nu }\right\}$ which is realized by the requirement ${\hat{g}}^{\mu \nu }{\hat{g}}_{\mu \nu }=4$. This result realizes, for CCG theory, the conceptual idea of a varying CC underlying unimodular gravity models proposed in previous studies. In the present setting, however, the formalism is undubious and acquires an immediate physical interpretation.

The result expressed by Equation (57) obtained in the classical framework of CCG theory is found to be consistent also with the expression of CC predicted by CQG theory. In particular, this concerns the functional dependence of $\Lambda \left(s\right)$, which is precisely of the type predicted by CQG theory as it follows from the canonical quantization of the gravitational field based of the classical Hamiltonian structure $\left\{x_{R},H_{R}\right\}$ [8,9]. More precisely, as shown in ref. [44] the quantum solution to the CC is found to be represented by a generally non-stationary function of proper time *s* of the form

(58) $$\Lambda_{CQG}\left(s\right)=\frac{\hbar^{2}}{{\left(\alpha L\right)}^{2}}\frac{1}{r_{th}^{4}}f\left(s\right),$$

with $\Lambda_{CQG}\left(s\right)$ to be denoted as CQG cosmological constant. Here, $\hbar^{2}$ is the reduced Planck constant and $r_{th}^{4}$ is a suitable dimensionless 4−scalar parameter estimated in ref. [9], which enters the prescription of the quantum probability density associated with the quantum state. In addition, f(s) is a strictly positive 4−scalar function of proper time determined by the equation

(59) $$f\left(s\right)\equiv p^{3}\left(s\right),$$

where in turn p(s) is a non-local 4−scalar function expressed by

(60) $$p\left(s\right)=\frac{1}{{\left(1+\frac{2}{\alpha L}\int_{s_{o}}^{s}ds^{\prime }a\left(s^{\prime }\right)\right)}^{1/2}}.$$

The analytical expression for the 4−scalar function a(s) can be found in ref. [44] and it follows from the solution of the quantum hydrodynamic equation for the phase-function associated with the quantum probability density through the Madelung decomposition. The prescription of the proper time functions f(s) and p(s) requires, therefore, the evaluation of the 4−scalar function a(s) [44]. However, qualitative properties can be inferred. In fact, in view of the prescription of the function p(s) it follows that its initial value occurring at $s=s_{o}=0$ is $p(s_{o})=1$, so that

(61) $$\Lambda_{CQG}\left(s_{o}\right)=\frac{\hbar^{2}}{{\left(\alpha L\right)}^{2}}\frac{1}{r_{th}^{4}}.$$

This implies that the relationship between $\Lambda_{CQG}\left(s\right)$ and $\Lambda_{CQG}\left(s_{o}\right)$ is

(62) $$\Lambda_{CQG}\left(s\right)=\Lambda_{CQG}\left(s_{o}\right)p^{3}\left(s\right).$$

Similarly, one can verify that in the limit s→+∞ the CC tends to a non-vanishing positive constant.

According to ref. [44], from the physical standpoint, $\Lambda_{CQG}\left(s\right)$ arises from space–time quantum-gravity contributions to the classical Einstein equations predicted by CQG theory when a trajectory-based representation of the related quantum wave equation is adopted in terms of the Generalized Lagrangian path formalism. The quantum solution for the CC was shown to be ascribed to the non-linear Bohm quantum vacuum interaction of the gravitational field with itself, namely produced by the self-interaction of massive gravitons, and to also generally depend on the realization of the quantum probability density for the quantum gravitational field tensor. This feature explains the dependence of $\Lambda_{CQG}\left(s\right)$ in Equation (58) in terms of the squared reduced Planck constant $\hbar^{2}$, which is the same type of dependence carried by the Bohm potential and ultimately due to the structure of the quantum-wave equation. The appearance of $\hbar^{2}$ therefore characterizes the solution for $\Lambda_{CQG}\left(s\right)$ as an intrinsically quantum term of the second order in *ℏ*, which retains the information of the non-linear quantum self-interaction of massive gravitons in a vacuum. The emerging physical picture predicts a generally non-stationary quantum cosmological constant $\Lambda_{CQG}\left(s\right)$ for which the explicit *s*-dependence arises because of the gradients (i.e., fluctuations) of the vacuum quantum gravitational energy density.

This outcome provides an alternative point of view for the physical interpretation of the evolution parameter *s* of the Hamiltonian structure underlying both CCG and CQG theories. Instead of identifying *s* only as the proper time defined along suitably synchronized classical geodesic curves associated with massive gravitons, here, a cosmological interpretation is given. The latter, in fact, can be associated with the intrinsic non-stationary character of the quantum-gravity generated cosmological constant $\Lambda_{CQG}$ with respect to its functional dependence on the same proper time *s*. The relationship is established through the bijections (57) and (58) which relate the 4−scalars *s* and $\Lambda_{CQG}$. The result realizes, for CCG and CQG theories, the project proposed in the literature for alternative quantum-gravity theories that underlies the concepts of relational evolution and evolving constants of motion. Accordingly, the dynamical evolution of the Hamiltonian dynamics in CCG and CQG theories can be measured not merely by the dynamical parameter *s*, but equivalently by the intrinsic change of the non-stationary quantum field $\Lambda_{CQG}$ that arises consistently from the quantum-gravity wave equation.

## 6. Conclusions

The theories of manifestly covariant classical and quantum gravity (CCG and CQG theories, respectively) are characterized by a unique Hamiltonian structure. Remarkably, the Hamilton equations provided by CCG theory admit a representation in evolution form in terms of a 4−scalar dynamical parameter *s*. This property is then inherited by CQG theory, whereby the same evolution form yields a non-stationary quantum-gravity wave equation. It must be stressed, however, that the dynamical evolution parameter identified by CCG and CQG theories is not related to a particular time coordinate stemming from some choice of reference frame or coordinate system implied by space–time slicing, as occurs, for example, in the well-known case of ADM formalism. From the physical point of view, the dynamical parameter *s* is associated with the arc length of suitably synchronized geodesic curves of massive gravitons in a background space–time. The evolution parameter identifies an observable quantum variable of CQG theory with associated conjugate momentum operator. As a notable feature, in fact, the same parameter can be associated with a non-stationary observable quantum cosmological constant predicted by CQG theory.

This provides an alternative point of view for the interpretation of the naturally evolving character of quantum gravity theory, to be equivalently set in terms of intrinsic change of the 4−scalar quantum cosmological constant. Notice, however, that such a dynamical behavior is not parametrized with respect to a particular coordinate-time in some coordinate reference frame. Instead, consistent with the principle of manifest covariance, it relies on the invariant proper time parameter *s* measured along appropriate geodesic curves that reach the cosmological horizon. This feature suggests a possible intriguing relationship between the quantum cosmological constant predicted by CQG theory and recently proposed models of cosmic evolution rooted on the idea of a varying dark energy, including a non-stationary cosmological constant supported by recent observations [45]. The dependence upon the parameter *s* might provide a novel route for the solution of the cosmological constant problem and the tension existing among different cosmological scenarios, a subject that is certainly worthy of further consideration in future studies.

These outcomes represent, for CCG and CQG theories, a possible solution to the so-called “problem of time” that affects physical and philosophical interpretations of alternative literature approaches to quantum gravity. The proof provided in the present research yields, in fact, an independent realization of the concepts of relational dynamics, evolving constants of motion and unimodular gravity that were proposed in previous studies as logical attempts or motivational explanations for alternative quantum-gravity theories. As such, the same result also represents a property of self-consistency of the theoretical setup underlying CCG and CQG theories, supporting their candidacy as promising frameworks for the establishment of canonical and manifestly covariant quantum gravity theory.

## Data Availability

Data are contained within the article.
